# Observations on Cholera Infantum

**Published:** 1848

**Authors:** J. H. McNutt

**Affiliations:** Annapolis, Indiana


### ARTICLE VII.

*Observations on Cholera Infantum.* By J. H. McNutt, M.D., of Annapolis, Indiana.

While the subject of fever has been a fruitful source of investigation, new theories having subverted the old dogmas of the schools, other subjects in our medical literature have not received that attention their importance demands—and in some small degree to supply that desideratum, the writer proposes to offer a few thoughts on infantile diseases — more particularly, on cholera infantum.

That cholera infantum is a disease of great fatality needs no argumentation. Condie says that it is a disease peculiar to the United States; and he further remarks that it prevails as an epidemic in the large cities of the United States during warm weather. Its cause and pathology are as yet not sufficiently known, as the basis on which to predicate a uniform and successful method of treatment. Children of from one to five years of age are the most obnoxious to its influence; but its principal subjects are those passing through their second summer or first dentition. Some have thought that the common causes of our autumnal fevers have an agency in the production of cholera infantum, but a moment's reflection will satisfy the inquirer after truth that this is not the fact. Cholera infantum is a disease of early snmmer, commencing before vegetation has commenced the process of decay, and therefore even the argument of "post hoc propter hoc" cannot be brought in aid of such an opinion. That dentition has the entire agency in its production is alike unsupported by facts and observation. Dentition is occurring during all periods of the year, whilst the ravages of cholera infantum are confined to the warmth of midsummer. Dentition, no doubt, is frequently an exciting cause, But the most rational conclusion is that the heat of summer is the principal cause, together with the change of diet that is consequent on that season of the year, as the tendency there is for the generation of acid in the prima via, in case of summer complaints of children, seems to prove. The stools are then acrid and generally fœtid, having a greenish appearance. There is, in the first place, a want of the proper assimilation of the ingesta. No doubt the heat of summer has an agency in debilitating the functions of digestion and assimilation. This being the primary pre-disposing cause, dentition and other accidental circumstances may act as exciting causes.

As to the pathology of cholera infantum, post obit examinations of those who have fallen victims to the disease have exhibited various lesions of the stomach and bowels. Early in the disease there is an anæmic condition of the stomach and bowels, with hyperæmia of the liver, but in the progress of the disease the stomach is characterized by increased redness, with points or patches of ulceration. Much more might be said of the *post mortem* appearance, but enough is known to satisfy any one that it is a disease of the alimentary canal, primarily of irritation, and consequently of increased action.

The symptoms are generally so well marked that they give a clear index to the disease, and therefore in most cases the diagnosis is not difficult. The pulse early in the disease is usually quick, small, and tense, with increased frequency. The invasion is often sudden, irritability of the stomach being among the first abnormal symptoms. So great is the disposition to vomit that every thing taken into the stomach is instantly ejected ; and indeed this constitutes one of the difficulties in the treatment of the disease, and to overcome it is the first and a very important indication in the treatment. The obstinate disposition to vomit sometimes occurs before the bowels become implicated in the disease, but most frequently in a short time, the bowels, sympathising with the stomach, commence running off. The stools at first are thin and watery, and often of a white color, containing very little feculent matter. Very often you will find in the stools undigested articles of diet the child may have eaten hours previously. The discharges generally assume a green appearance, and if the disease is not broken up in time, there is in the latter period of the disease bloody stools of a dysenteric character. So much for the appearance of the alvine evacuations in the early stage of cholera infantum.

There is often not much fever. The face is generally pale, skin dry, with shrunken aspect of the countenance, the patient choosing to lay undisturbed, moving only as the calls of nature interrupt the quiet. But as the disease advances, other functions become implicated in the morbid train of derangements. The fever assumes a continuous form, no doubt depending on some local cause, either on intestinal inflammation of a subacute character, or, which is doubtless more frequently the case, on an inflamed condition of the mesenteric glands. The thirst increases, the mouth and fauces become dry, the eyes dull and heavy, and profound coma frequently closes the scene. The prognosis in the disease should always be given with caution. It is a disease, when treated early and promptly, that is, perhaps, as amenable to curative means as any of the formidable diseases of childhood. But, when suffered to progress for a few days without treatment, or, what is worse, maltreated, we may expect an unfavorable termination. When the stools become more natural, the irritability of the stomach is quieted, the thirst abated, we may pronounce more favorably.

Thc treatment of the disease is a matter of the most importance in its history. The previous health, habits, &c., of the child should be taken into account. The indications in the treatment of cholera infantum are to allay the irritability of the stomach, to relieve congestion of the liver, and to determine to the surface. The first indication is generally filled by the exhibition of a few grs. of sub-mur. hyd. The following is a valuable formula, viz: sub-mur. hyd., grs. 3; sub. carb. soda, grs. 6; mix and divide into three parts, one of which should be given every two hours. If this should fail to quiet the irritability of the stomach, a sinapism may be laid over the epigastric region. The protracted use of calomel in the diseases of children cannot be too strongly condemned. Calomel, after it has filled the indications we intended by its administration, should be laid aside, and other therapeutic means used. I have seen the little patients doomed daily to swallow the alteratives of calomel, (by the routinist,) merely because the stools are green. Now, before we push our mercurials too far, we should stop one moment and inquire the cause of the green appearance of the stool. It is known to all that to give a child milk or any other article of diet that will coagulate by the admixture with acid, that it will immediately coagulate when received into the stomach of a child who is passing green stools. Every close observer of the diseases of childhood must have noticed this. Now, the indications are clearly to overcome the acidity of the stomach, and give tone to the digestive functions. I have known nothing answer a more valuable purpose than the following syrup, viz: take of rhubarb burnt, 2 ʒ; boiling water, 8 ℥; white or loaf sugar, 2 ʒ; boil for a few moments, then strain; to each ℥ of this syrup, after straining, add 5 grs. of the sub-carb. of soda.* It will be seen that the syrup here directed is nearly the same as the rhubarb syrup of the shops, except the burning of the rhubard, and the addition of the alkali, which I consider a valuable improvement. Now my plan of treating cholera infantum is, in the first place, if there is torpor of the liver to premise the treatment by the administration of a few grs. of calamel, as above indicated, after the operation of which commence the use of the syrup. To a child two years old, a tea-spoonful three or four times each day, other ages in proportion. If the bowels are inclined to run off too much, add a few drops of paregoric or Godfrey's cordial to the first few doses. Should there be cerebral symptoms, and you fear the administration of an opiate to restrain the action of the bowels, give astringents. The vegetable astringents are the best, such as the tinc. kino or catechu, or what is perhaps better than any other astringent, geraneum maculatum, in tincture or infusion. It may be given alone or in conjunction with the syrup. Should the fever attendant on the disease be of an intermittent character, the apyrexia should be filled by the administration of the sulphate of quinine in doses pro re nati, in protracted cases. When there is nervousness, much benefit may be had from the use of the precipitated carb. ferri. Take of precip. carb. ferri, 1 ʒ; port wine, 4 ℥; add the ferri to the wine. Of this a tea-spoonful may be given three or four times each day, with a proper diet and wholesome air. Such is a brief synopsis of a treatment with which I have abundant reason to be satisfied.

I would again repeat, let no one continue in the persistent use of calomel, to the exclusion of other means, when he has

* In a note in No. 3 of Braitewaite's Retrospect, page 60, the reader will find the medicinal virtues of burnt rhubarb, by E. P. Hoblyn, of the Middlesex Hospital. "It may be useful to the profession to know the value of burnt rhubarb in diarrhœa. I have used it for seven years, and found it more serviceable, in diarrhœa attendant on the last stage of consumption, than the chalk mixture and opium, or any other of the usual remedies I have known it used with the same pleasing effects for more than 20 years. In accidental diarrhœa, after one or two doses, the pains quickly subside, and the bowels return to their natural state. The dose is from five to ten grains. The manner of preparing is to burn the rhubarb in an iron crucible, stirring it until it is blackened, then smother it in a covered jar. It loses two-thirds of its weight by incineration, and is nearly tasteless. In no case where I have known it given, has it failed. I have given it in port wine, milk, or water."

no other criterion for its administration but the appearance of green stools. If he does he will find patients rapidly sinking to a premature grave. I would not be understood as condemning the proper use of calomel; on the contrary, I believe it to be a valuable means, and in all cases where there is bilious vomiting and fever, the treatment, as before remarked, should be premised by the administration of calomel in appropriate and at suitable periods, aided by the other adjuvants above indicated.

Much might be said of prophylactics. Let every one who has the care of children, avoid as much as possible all the exciting causes, such as errors in diet, unwholesome air, and difficult dentition.

